# Insight into the Role of Surface Wettability in Electrocatalytic Hydrogen Evolution Reactions Using Light-Sensitive Nanotubular TiO_2_ Supported Pt Electrodes

**DOI:** 10.1038/srep41825

**Published:** 2017-02-06

**Authors:** Chenhui Meng, Bing Wang, Ziyue Gao, Zhaoyue Liu, Qianqian Zhang, Jin Zhai

**Affiliations:** 1 Key Laboratory of Bio-Inspired Smart Interfacial Science and Technology of Ministry of Education, Beijing Key Laboratory of Bio-inspired Energy Materials and Devices, School of Chemistry and Environment, Beihang University, Beijing 10019, China; 2 School of Physics and Nuclear Energy Engineering, Beihang University, Beijing 100191, China

## Abstract

Surface wettability is of importance for electrochemical reactions. Herein, its role in electrochemical hydrogen evolution reactions is investigated using light-sensitive nanotubular TiO_2_ supported Pt as hydrogen evolution electrodes (HEEs). The HEEs are fabricated by photocatalytic deposition of Pt particles on TiO_2_ nanotubes followed by hydrophobization with vaporized octadecyltrimethoxysilane (OTS) molecules. The surface wettability of HEEs is subsequently regulated *in situ* from hydrophobicity to hydrophilicity by photocatalytic decomposition of OTS molecules using ultraviolet light. It is found that hydrophilic HEEs demonstrate a larger electrochemical active area of Pt and a lower adhesion force to a gas bubble when compared with hydrophobic ones. The former allows more protons to react on the electrode surface at small overpotential so that a larger current is produced. The latter leads to a quick release of hydrogen gas bubbles from the electrode surface at large overpotential, which ensures the contact between catalysts and electrolyte. These two characteristics make hydrophilic HEEs generate a much high current density for HERs. Our results imply that the optimization of surface wettability is of significance for improving the electrocatalytic activity of HEEs.

Hydrogen, as a clean and portable energy carrier, has been attracting worldwide attention because of its possibility to replace fossil fuels in the future^1^,^2^. Electrocatalytic water splitting is one of sustainable route to produce hydrogen from water by electricity^3^,^4^. In this route, catalytic Pt electrodes are often used as hydrogen evolution electrodes (HEEs) to achieve hydrogen evolution reactions (HERs)^5^,^6^. A complete HER in acidic solution involves three procedures of proton migration, proton reduction and hydrogen gas release^7^. The last two procedures occur at solid-liquid (electrode-electrolyte) and solid-gas (electrode-hydrogen) interface respectively, which affect the electrocatalytic activity of HEEs significantly. Therefore, understanding the role of surface properties of HEEs in HERs is of significance for improving their electrocatalytic performances.

Surface wettability, as one of fundamental surface properties of an electrode, shows a significant influence on electrolyte-wetting, redox electron transfer and gas release in electrochemical reactions^8^,^9^,^10^,^11^. For example, a superhydrophobic surface can control the precursor-wetting and guide gold nanoparticles to electrochemically grow along solid-liquid-gas triphase interface^9^. A hydrophilic surface enhances the extracellular electron transfer rate between microbes and electrode^10^. In water electrolysis under microgravity, the oxygen evolution is also affected significantly by the wettability of electrode^11^. However, to the best of our knowledge, how the surface wettability of HEEs affects the HERs has not been investigated systematically.

Recently, lots of progress has been achieved on the wettability regulation of solid surface using responsive materials sensitive to external stimuli^12^,^13^,^14^,^15^,^16^, which makes it possible to regulate the surface wettability of HEEs in a wide range. Herein, the surface wettability of HEEs consisting of octadecyltrimethoxysilane-modified nanotubular TiO_2_ supported Pt electrodes (Pt-TiO_2_-NTs) was regulated *in situ* from hydrophobicity to hydrophilicity using external stimulus of ultraviolet (UV) light and its role in HERs was investigated systematically. It is found that hydrophilic HEEs generate a much higher current density for HERs when compared with hydrophobic ones. We conclude that this high electrocatalytic activity is associated with their large electrochemical active area of Pt and low adhesion force to a gas bubble.

## Results and Discussion

**Figure 1:**
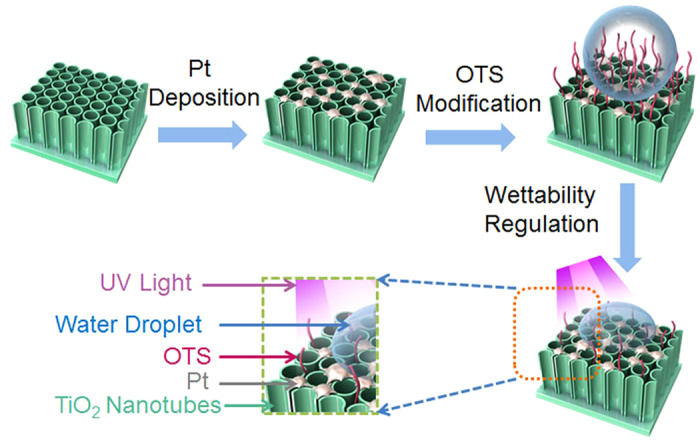
Flow chart for the fabrication and wettability regulation of nanotubular TiO_2_ supported Pt electrodes (Pt-TiO_2_-NTs). Pt catalysts were deposited on the surface of anodized TiO_2_ nanotubes (TiO_2_-NTs) by photocatalytic reduction, which was followed by hydrophobization with vaporized octadecyltrimethoxysilane (OTS) molecules. The wettability of Pt-TiO_2_-NTs was subsequently regulated *in situ* from hydrophobicity to hydrophilicity using ultraviolet (UV) light.

Electrochemically anodized TiO_2_ nanotubes (TiO_2_-NTs) on Ti metals have been used as a substrate to support electrocatalysts of Pt or MoS_2_ for HERs^17^,^18^. The significant advantage of TiO_2_-NTs as a substrate is that their photocatalysis can trigger some special reduction reactions, which result in the direct loading of particulate catalysts on the surface of TiO_2_-NTs without the necessity of using additional immobilization procedures. Furthermore, TiO_2_-NTs shows a rough surface, which can be used to fabricate hydrophobic surface via chemical modification^19^,^20^,^21^,^22^,^23^. As shown in Fig. 1, the fabrication and wettability regulation of Pt-TiO_2_-NTs involves four steps: (1) formation of anatase TiO_2_-NTs on Ti metals by a combination of electrochemical anodization and high-temperature calcination^24^,^25^,^26^,^27^,^28^; (2) deposition of Pt catalysts on TiO_2_-NTs by photocatalytic reduction of chloroplatinic acid under UV illumination^29^; (3) hydrophobization with vaporized octadecyltrimethoxysilane (OTS) molecules^19^,^20^,^21^,^22^,^23^; (4) wettability regulation of OTS-modified Pt-TiO_2_-NTs by photocatalytic decomposition of OTS molecules using UV light^19^,^20^,^22^,^23^.

**Figure 2:**
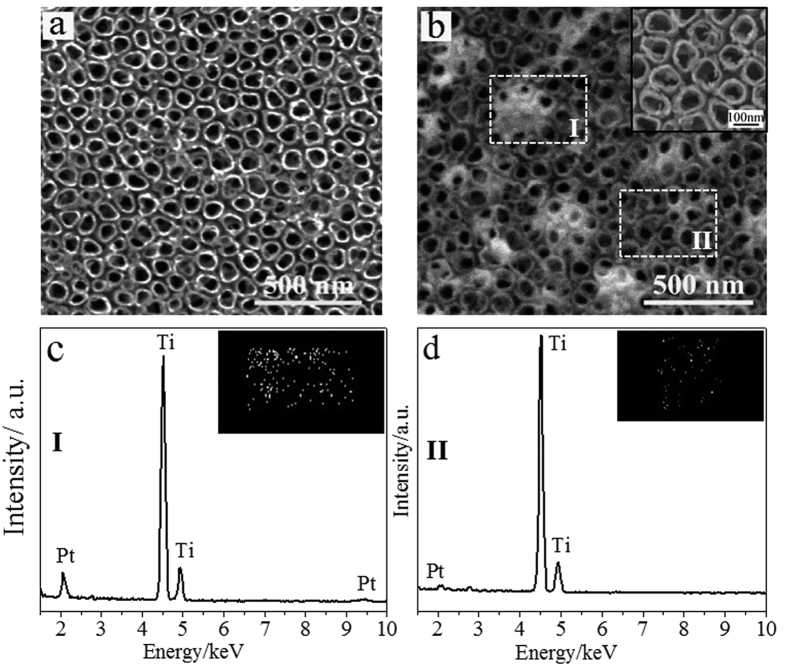
(**a,b**) SEM images of TiO_2_-NTs (**a**) before and (**b**) after photocatalytic deposition of Pt particles. Only a small quantity of TiO_2_-NTs is covered by Pt aggregates with a size of ~230 nm. Inset of b: magnified SEM image of TiO_2_-NTs without covering of Pt aggregates. Pt nanoparticles with a size of ~10 nm distribute on the perimeter of nozzles of TiO_2_-NTs. (**c,d**) Element maps and EDX patterns on the specific areas (**c**) with and (**d**) without covering of Pt aggregates as shown in b (area I and II). In both areas, the characteristic lines at energy of ~2.05 keV for Pt elememts are detected. The area with covering of large aggregates demonstrates a high Pt content.

As shown in Fig. 2a, the as-prepared TiO_2_-NTs show a clean nanopore surface with a pore inner diameter of ~86 nm and a pore wall of ~11 nm. After photocatalytic deposition of Pt (Fig. 2b), only a small quantity of TiO_2_-NTs is covered by Pt aggregates with a size of ~230 nm. Most of TiO_2_-NTs are not covered by Pt aggregates. The magnified SEM image of TiO_2_-NTs without covering of Pt aggregates (Inset of Fig. 2b) show that Pt nanoparticles with a size of ~10 nm distribute on the perimeter of nozzles of TiO_2_-NTs, which indicates that Pt catalysts distributes throughout the whole surface of TiO_2_-NTs. Our results are consistent with the findings of Herrmann *et al*. that some Pt particles are present as large aggregates after a long-time photocatalytic reaction besides the small Pt nanoparticles^30^. Element maps and energy dispersive X-ray microanalysis (EDX) patterns are used to further investigate the distribution of Pt catalysts on the surface of TiO_2_**-**NTs. Figure 2c and d show the element maps and EDX of two specific areas with and without covering of Pt aggregates (area I and II in Fig. 2b respectively). In both areas, the characteristic lines at energy of ~2.05 keV for Pt elememts are detected^31^,^32^, which indicates that Pt elements distribute throughout the whole surface of TiO_2_-NTs. The area consisting of large aggregates demonstrates a higher Pt content when compared with the one without aggregates, which is evidenced by the strong Pt characteristic lines in Fig. 2c. The composite surface of Pt-TiO_2_-NTs demonstrates one important characteristic that the surface of TiO_2_-NTs is not densely covered by Pt particles. The exposed TiO_2_-NTs surface is necessary for wettability regulation using UV light because photocatalytic oxidation ability is available only on TiO_2_ surface.

**Figure 3:**
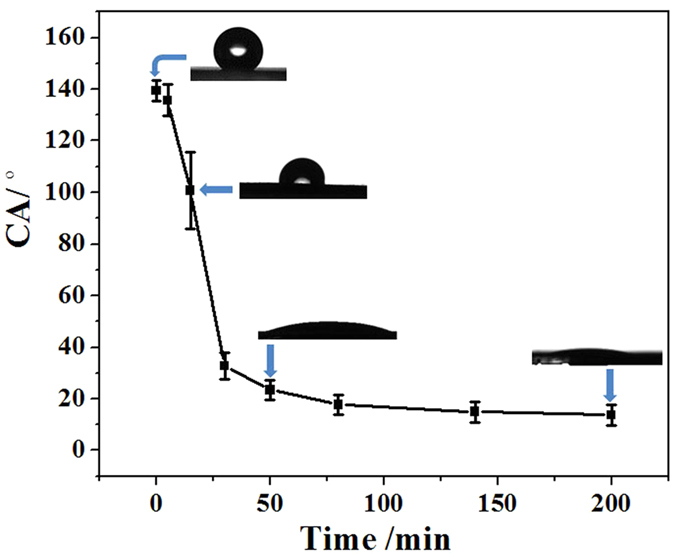
The evolution of water contact angles (CAs) of OTS modified Pt-TiO_2_-NTs following *in situ* UV illumination. Insets: The corresponding water CAs when the UV illumination time is 0, 15, 50 and 200 min. The water CAs are sensitive to light, which can be regulated on demand from ~139° to ~13°.

In order to enable the surface wettability of Pt-TiO_2_-NTs to be sensitive to UV light, OTS molecules were deposited on their surface using chemical vapor deposition method (Fig. 1). After OTS modification, as shown in Fig. 3, the surface of Pt-TiO_2_-NTs is hydrophobic with a water contact angle (CA) of ~139°. This hydrophobicity is ascribed to the synergistic effect of rough nanopore surface and hydrophobic octadecyl carbon chains in OTS molecules^33^,^34^,^35^. It has been known that the surface hydroxyls of TiO_2_-NTs are important for the silanization reactions because they can form covalent bonds with OTS molecules^36^,^37^. However, in order to immobilize silane molecules on the surface of Pt, an anodization process to generate oxide surface with hydroxyls is necessary^38^,^39^. Therefore, it is reasonably considered that OTS molecules are difficult to covalently bond to the as-prepared Pt particles (including the large Pt aggregates with a size of ~230 nm and the small Pt nanoparticles with a size of ~10 nm). In order to confirm the distribution of OTS molecules, the silicon EDX patterns of OTS-modified Pt-TiO_2_-NTs after UV illumination for 200 min (Figure S1 in Supplementary information) are measured. It has been known that after a photocatalytic reaction the carbon chains of OTS molecules will be decomposed and silicon elements are left^40^. Figure S1 indicate clearly that Si elements mainly distribute on the surface of TiO_2_-NTs. However, on the surface of Pt aggregates, the characteristic line of Si element is very weak. Our results indicated that OTS molecules can deposit selectively on TiO_2_-NTs. Following *in situ* UV illumination, the water CA can be controlled on demand from ~139° to ~13°, which are determined by the UV illumination time. The decrease in water CA is ascribed to wettability change of TiO_2_-NTs, which results from the controllable decomposition of alkyl chains in OTS molecules by the oxidation ability of TiO_2_ photocatalysis^40^,^41^.

**Figure 4:**
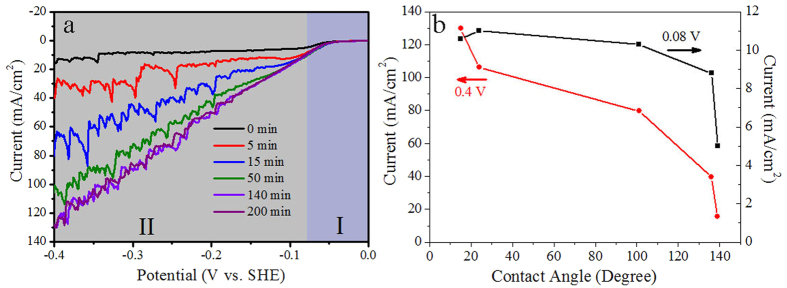
(**a**) Polarization curves of OTS-modified Pt-TiO_2_-NTs after *in situ* UV illumination for 0, 5, 15, 50, 140 and 200 min in 0.5 M H_2_SO_4_. The polarization curves are divided into two regions of I and II by a potential of −0.08 V. (**b**) The quantitative correlations between the water contact angles (CAs) of OTS-modified Pt-TiO_2_-NTs and the catalytic current density for HERs at an overpotential of 0.08 V and 0.4 V. Following the decrease of water CA by UV illumination, the enhanced degree of current density in region II is much larger than that in region I.

Subsequently, we investigated the role of surface wettability of Pt-TiO_2_-NTs in HERs. The wettability was controlled *in situ* by UV illumination time. Figure 4a shows the polarization curves of Pt-TiO_2_-NTs with different wettability in 0.5 M H_2_SO_4_ electrolyte. The quantitative correlations between the water CAs of Pt-TiO_2_-NTs and the cuerrent density values for HERs at an overpotential of 0.08 V and 0.4 V are summarized in Fig. 4b. The polarization curves are divided into two regions of I and II by a potential of −0.08 V (vs. SHE). For hydrophobic Pt-TiO_2_-NTs with a water CA of ~139°, the characteristics of polarization curve in region I and II are different. In region I with overpotential smaller than 0.08 V, the increase in the overpotential enhances the current density for HERs (Fig. 4a and Figure S2 in Supplementary information). In this region, it is considered that the evolved hydrogen gas can dissolve into the electrolyte because a small current density is not sufficient to supersaturate the electrolyte^42^. Therefore, no hydrogen bubbles form on the surface of Pt-TiO_2_-NTs. The current density for HERs is only dependent on the potential. At a typical potential of −0.08 V (vs. SHE), hydrophobic Pt-TiO_2_-NTs generate a current density of 5.0 mA/cm^2^. However, in region II with overpotential larger than 0.08 V, the concentration of hydrogen gas in the electrolyte reaches a critical value, the hydrogen gas bubbles nucleate and grow on the surface of electrode^42^, which will occupy the catalytically active sites of Pt for HERs. Therefore, in region II, although the overpotential increases from 0.08 to be 0.4 V (vs. SHE), the current density increases very slowly from 5.0 to 15.6 mA/cm^2^ for hydrogen evolution.

When the water CA of hydrophobic Pt-TiO_2_-NTs is lowered by UV illumination, the current density of Pt-TiO_2_-NTs in region I increases as shown in Fig. 4b and Fig. S2 in supplementary information. Hydrophilic Pt-TiO_2_-NTs with a water CA of ~24° obtained by UV illumination for 50 min generate a current density of 11.0 mA/cm^2^ at −0.08 V (vs. SHE), which is almost 2.2 times as much as that (5.0 mA/cm^2^) of hydrophobic ones with a water CA of ~139°. Further extending the UV illumination time does not enhance the current density in region I (Fig. 4b). Since no large hydrogen gas bubbles are generated in region I because of the small overpotential, the enhanced current density is ascribed to the increase in the electrochemical active area of Pt (ECA) by a hydrophilic surface. During wettability regulation, the ECA of large Pt aggregates remains almost unchanged because they stay on the surface of TiO_2_-NTs and can contact the electrolyte. However, the ECA of the small nanoparticles (~10 nm) on the perimeter of nozzles of TiO_2_-NTs is related with the surface wettability. For a hydrophobic surface, Pt nanoparticles cannot be wetted by the electrolyte because of the sealed air in the nanotubes, which results in a small ECA. When the surface wettability of Pt-TiO_2_-NTs is regulated to be hydrophilic, TiO_2_-NTs can be wetted by the electrolyte. Pt nanoparticles on the perimeter of nozzles of TiO_2_-NTs then contact the electrolyte and show a large ECA.

**Figure 5:**
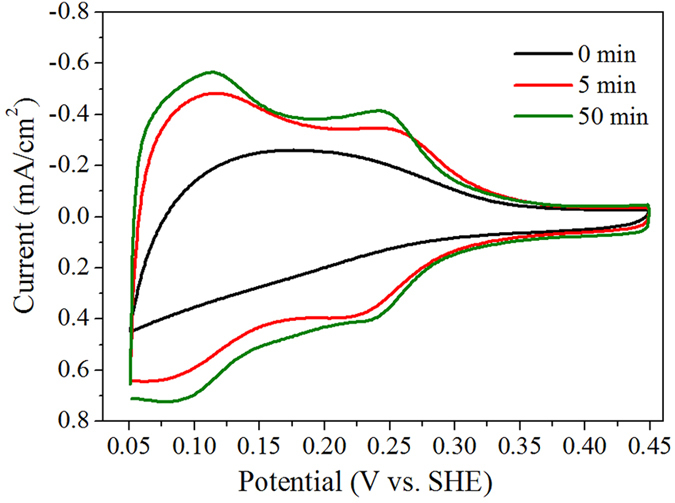
Cyclic voltammogram curves of hydrophobic OTS-modified Pt-TiO_2_-NTs in 0.5 M H_2_SO_4_ after UV illumination for 0, 5 and 50 min. The scan rate is 50 mV/s. The two couples of current peaks are associated with the hydrogen adsorption and desorption. The calculated electrochemical active area based on hydrogen adsorption increases following the increase of UV illumination time.

The ECA of Pt can be estimated based on the adsorption and desorption of hydrogen as electrochemical adsorbates. The hydrogen adsorption charge is then converted into ECA based on the well-established relationship of 210 μC/cm^2^ for polycrystalline Pt surfaces^43^,^44^. As shown in Fig. 5, hydrophobic Pt-TiO_2_-NTs with a water CA of ~139° demonstrate a low ECA of ~4.9 cm^2^. After UV illumination for 5 and 50 min, two couples of current peaks are observed for hydrogen adsorption and desorption. The calculated ECA of Pt based on hydrogen adsorption is ~8.7 and ~9.9 cm^2^ respectively. Therefore, hydrophilic Pt-TiO_2_-NTs allow more protons to react on the electrode surface, which contributes to the enhancement of current density for HERs in region I of Fig. 4a.

**Figure 6:**
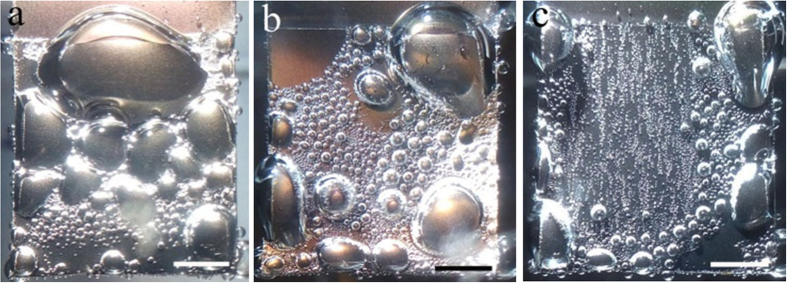
Formation of hydrogen gas bubbles on the surface of OTS-modified Pt-TiO_2_-NTs electrode at −0.4 V (vs. SHE) after UV illumination for (**a**) 0, (**b**) 50 and (**c**) 200 min. The hydrophilicity reduces the size of hydrogen gas bubbles sticking on the electrode surface. The scale bar is 250 mm.

However, in region II with large overpotential, the current density is enhanced exceptionally by a hydrophilic surface. As shown in Fig. 4b, Hydrophilic Pt-TiO_2_-NTs with a water CA of ~15° obtained by UV illumination for 140 min generate a current density of 129.8 mA/cm^2^ at −0.4 V (vs. SHE), which is almost nine times as much as that of hydrophobic Pt-TiO_2_-NTs with a water CA of ~139° (15.6 mA/cm^2^). The different degree for current enhancement by a hydrophilic surface at small (0.08 V) and large (0.4 V) overpotential indicates that the surface wettability is not the only reason for the high catalytic activity of hydrophilic Pt-TiO_2_-NTs. In region II, a lot of hydrogen gas bubbles are generated during HERs because of the large overpotential. Therefore, it is considered that this exceptionally enhanced current density in region II by a hydrophilic surface should be related with its low adhesion force of electrode surface to a gas bubble. In HERs, when the concentration of hydrogen in electrolyte reaches a critical value, hydrogen gas bubbles will nucleate and grow on the electrode surface, which will occupy the catalytically active sites. Once the surface adhesive force cannot sustain the hydrogen gas bubbles, they will detach the electrode surface and releases the occupied active sites for HERs^42^. As shown in Fig. 6a, for hydrophobic Pt-TiO_2_-NTs with water CA of ~139°, when the potential is scanned to be −0.4 V (vs. SHE), a lot of hydrogen gas bubbles with a size larger than ~80 mm adhere on the electrode surface. The video during polarization process demonstrates that the small hydrogen bubbles will merge into a large one, which sticks on the electrode surface strongly (Video S1 in Supplementary information). The smooth polarization curve of Pt-TiO_2_-NTs with a water CA of ~139° (Fig. 4a) also imply that the hydrogen gas bubble is difficult to leave the surface of hydrophobic electrode. These gas bubbles prevent the contact between catalysts and electrolyte, and consequently reduce the electrocatalytic activity. After the hydrophobic Pt-TiO_2_-NTs are illuminated by UV light for 50 min, some large gas bubbles will leave the electrode surface in HERs. The size of the adhered gas bubbles decreases obviously (Fig. 6b), which reduces the occupation area of gas bubbles and increases the catalytic activity. The large current shake in Fig. 4a also implies the detachment of gas bubbles from the electrode surface. The size of adhered bubbles can be further decreased by extending the UV illumination time to be 200 min (Fig. 6c). In this case, most of small hydrogen bubbles can leave the electrode surface quickly during hydrogen evolution (Video S2 in Supplementary information), which results in a high electrocatalytic activity^45^,^46^,^47^.

**Figure 7:**
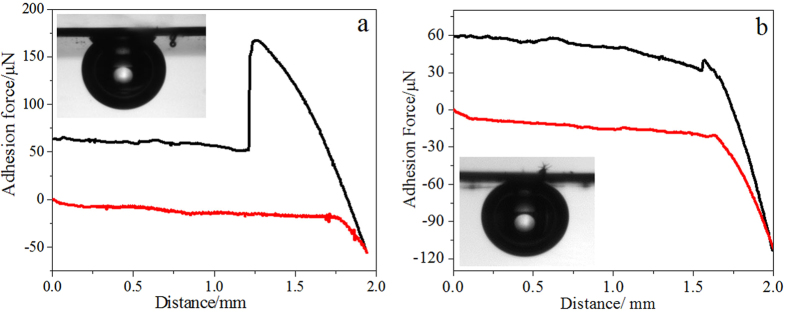
Adhesive force-distance curves when Pt-TiO_2_-NTs with a water CA of ~139° (**a**, hydrophobic) and ~14° (**b**, hydrophilic) approach to and retract from an external gas bubble. Insets: The corresponding underwater gas CAs. A hydrophilic surface shows a low adhesion force and a high underwater gas CA.

It has been well-known that the adhesion force of a solid surface to a gas bubble is related with its surface wettability^48^,^49^. Herein, a high-sensitivity microelectromechanical balance system is used to measure quantitatively the adhesion force of Pt-TiO_2_-NTs with different wettability to a gas bubble in water. Figure 7a shows the adhesive force-distance curves when Pt-TiO_2_-NTs with a water CA of ~139° approach to and retract from a gas bubble with a volume of 5 μL. The starting position of Pt-TiO_2_-NTs is defined to be zero point of distance. As shown in Fig. 7a, at a distance of ~1.95 mm, Pt-TiO_2_-NTs contact the gas bubble. Then, Pt-TiO_2_-NTs start to move away from the gas bubble. The balance force between Pt-TiO_2_-NTs and gas bubble gradually increases because of the adhesion of gas bubble on the surface of Pt-TiO_2_-NTs, which reaches a maximum at a distance of ~1.26 mm. When the gas bubble breaks away from Pt-TiO_2_-NTs at the distance of ~1.26 mm, the force between Pt-TiO_2_-NTs and gas bubble disappears. The jump at distance of ~1.26 mm represents the the adhesive force of Pt-TiO_2_-NTs to a gas bubble, which is calculated to be ~116 μN. A gas bubble is easy to adhere onto the surface of hydrophobic Pt-TiO_2_-NTs (Video S3 in Supplementary information). This large adhesion force is ascribed to the existence of trapped air pockets in TiO_2_-NTs by water. When an external gas bubble approach to the surface area of hydrophobic Pt-TiO_2_-NTs, it will coalesce with the air pockets in TiO_2_-NTs to form fresh gas-liquid-solid three-phase lines (as evidenced by a relatively low underwater gas CA of ~136° in the inset of Fig. 7a), which results in a large adhesion force^48^. After the hydrophobic Pt-TiO_2_-NTs are converted to be hydrophilic by UV illumination, as shown in Fig. 7b, the small jump at a distance of 1.57 mm represents a small adhesive force of ~9 μN because of the complete wetting of TiO_2_-NTs by water and no air is trapped. The surface therefore demonstrates a high underwater gas CA of ~157° (inset of Fig. 7b). A gas bubble is very hard to adhere onto the surface of hydrophilic Pt-TiO_2_-NTs (Video S4 in Supplementary information). This extremely low adhesion force can drive large hydrogen gas bubbles to leave the electrode surface quickly in HERs which ensures the contact between Pt catalytic active sites and electrolyte, and increases the catalytic activity.

## Conclusions

In summary, the role of surface wettability in HERs has been investigated using light-sensitive hydrophobic Pt-TiO_2_-NTs as HEEs. The surface wettability of Pt-TiO_2_-NTs is facilely regulated *in situ* from hydrophobicity to hydrophilicity using an irradiation of UV light. The measurements of polarization curves indicate that the electrocatalytic activity of Pt-TiO_2_-NTs for HERs is enhanced by a hydrophilic surface. At a low overpotential without formation of large hydrogen bubbles, this enhancement of current density mainly results from the increased electrochemical active area. However, at a high overpotential, the exceptionally enhanced current density mainly results from the low adhesion force of a hydrophilic surface for a gas bubble, which can drive large hydrogen gas bubbles to leave the electrode surface quickly and ensures the contact between Pt catalytic and electrolyte. Our present work indicates that the optimization of surface wettability of HEEs is important for the improvement of their electrocatalytic activity for HERs.

## Experimental

### Fabrication of nanotubular TiO_2_ supported Pt electrodes

TiO_2_ nanotubes (TiO_2_-NTs) on Ti substrate were prepared by an electrochemical anodization at a constant voltage of 20 V for 20 min in 0.5% HF aqueous solution^20^,^21^,^22^,^23^. The anatase crystallization was achieved by annealing as-formed TiO_2_-NTs at 450 °C for 3 h. Pt particles were deposited directly on TiO_2_-NTs by photocatalytic reduction of chloroplatinic acid (H_2_PtCl_6_·6H_2_O) precursor in H_2_O/isopropanol mixed solvent with volume ratio of 9:1^29^. The photocatalytic reaction time is 50 min. The light source is 365-nm ultraviolet (UV) light from a high-pressure mercury lamp with an irradiance of 5 mW/cm^−2^. For convenience, Pt-deposited TiO_2_-NTs were denoted as Pt-TiO_2_-NTs.

### *In situ* Wettability Regulation of Pt-TiO_2_-NTs Using UV Light

In order to enable the surface wettability of Pt-TiO_2_-NTs to be sensitive to external UV light, the surface was hydrophobicated with octadecyltrimethoxysilane (OTS, Aldrich) molecules. Pt-TiO_2_-NTs were placed in a Teflon-lined stainless autoclave with 100 μg of OTS, which was heated to 130 °C and kept at this temperature for 3 h. Then, the surface wettability of OTS-modified Pt-TiO_2_-NTs was regulated *in situ* from hydrophobicity to hydrophilicity gradually using UV illumination^19^,^20^,^22^,^23^. The strong oxidation ability of TiO_2_ photocatalysis under UV illumination decomposed OTS molecules, which resulted in a wettability change.

### Evaluation of Electrocatalytic Activity

The electrocatalytic activities of Pt-TiO_2_-NTs with different wettability for HERs were evaluated by measuring the polarization curves in 0.5 M H_2_SO_4_ solution using a three-electrode configuration^17^. The polarization potential was supplied by a CHI660D potentiostat (Shanghai Chenhua Apparatus Co., China) with a scanning rate of 5 mV/s. The working electrode is Pt-TiO_2_-NTs with a geometric area of 1 cm^2^, and the counter electrode is Pt wire. The electrochemical active area of Pt was calculated by measuring the cyclic voltammogram curves of Pt-TiO_2_-NTs electrodes in 0.5 M H_2_SO_4_ solution using a scan rate of 50 mV/s. An Ag/AgCl electrode in 3.5 M KCl solution was used as a reference electrode. The potential was calibrated to be against standard hydrogen electrode (SHE) based on an equation of φ (V vs. SHE) = φ (V vs. Ag/AgCl) + 0.205 V.

### Characterizations

The morphology of Pt-TiO_2_-NTs was observed using an environmental scanning electron microscope (FEI Quanta FEG 250) at 10 kV. An OXFORD INCA Energy 250 energy spectrum analyzer linked on JEOL JSM-7500F field-emission scanning electron microscope was used to detect the surface chemical elements. The evolution of hydrogen gas bubbles during HERs was imaged *in situ* by a camera. The water and underwater gas bubble contact angles (CAs) of the electrode were characterized using an OCA20 contact-angle system (Dataphysics, Germany). The adhesive force of the electrode surface to a gas bubble was measured using a high-sensitivity micro-electromechanical balance system (Dataphysics DCAT21, Germany). A gas bubble with a volume of 5 μL was suspended on a metal ring, and then Pt-TiO_2_-NTs began to approach to the gas bubble. The starting position of Pt-TiO_2_-NTs is defined to be zero point of distance. Once contacted, Pt-TiO_2_-NTs started to move away from the gas bubble. The balance force between Pt-TiO_2_-NTs and gas bubble would gradually increase, and reach a maximum when the gas bubble just broke away from Pt-TiO_2_-NTs. The change of balance force resulting from the detachment of gas bubble from the surface of Pt-TiO_2_-NTs was defined as the adhesive force.

## Additional Information

**How to cite this article**: Meng, C. *et al*. Insight into the Role of Surface Wettability in Electrocatalytic Hydrogen Evolution Reactions Using Light-Sensitive Nanotubular TiO_2_ Supported Pt Electrodes. *Sci. Rep.* **7**, 41825; doi: 10.1038/srep41825 (2017).

**Publisher's note:** Springer Nature remains neutral with regard to jurisdictional claims in published maps and institutional affiliations.

## Supplementary Material

Supplementary Information

Supplementary Video S1

Supplementary Video S2

Supplementary Video S3

Supplementary Video S4

## References

[b1] Schlapbach L. & Züttel A. Hydrogen-storage materials for mobile applications. Nature 414, 353–358 (2001). 11713542 10.1038/35104634

[b2] Turner J. A. Sustainable hydrogen production. Science 305, 972–974 (2004). 15310892 10.1126/science.1103197

[b3] Vrubel H., Merki D. & Hu X. Hydrogen evolution catalyzed by MoS_3_ and MoS_2_ particles. *Energy Environ*. Sci. 5, 6136–6144 (2012).

[b4] Laursen A. B., Kegnæs S., Dahl S. & Chorkendorff I. Molybdenum sulfides—efficient and viable materials for electro- and photoelectrocatalytic hydrogen evolution. *Energy Environ*. Sci. 5, 5577–5591 (2012).

[b5] Popczun E. J., Read C. G., Roske C. W., Lewis N. S. & Schaak R. E. Highly active electrocatalysis of the hydrogen evolution reaction by cobalt phosphide nanoparticles. Angew. Chem. Int. Ed. 53, 5427–5430 (2014). 10.1002/anie.201402646 24729482

[b6] Merki D., Fierro S., Vrubel H. & Hu X. Amorphous molybdenum sulfide films as catalysts for electrochemical hydrogen production in water. Chem. Sci. 2, 1262–1267 (2011).

[b7] Gu C., Norris B. C., Fan F. F., Bielawski C. W. & Bard A. J. Is base-inhibited vapor phase polymerized PEDOT an electrocatalyst for the hydrogen evolution reaction? exploring substrate effects, including Pt contaminated Au. ACS Catal. 2, 746−750 (2012).

[b8] Wu Y., Liu K., Su B. & Jiang L. Superhydrophobicity-mediated electrochemical reaction along the solid-liquid-gas triphase interface: edge growth of gold architectures. Adv. Mater. 26, 1124–1128 (2014). 24243745 10.1002/adma.201304062

[b9] Ding C., Lv M., Zhu Y., Jiang L. & Liu H. Wettability-regulated extracellular electron transfer from the living organism of shewanella loihica PV-4. Angew. Chem. Int. Ed. 54, 1446–1451 (2015). 10.1002/anie.201409163 25470810

[b10] Sakuma G., Fukunaka Y. & Matsushima H. Nucleation and growth of electrolytic gas bubbles under microgravity. Int. J. Hydrogen Energy 39, 7638–7645 (2014).

[b11] Stoerzinger K. A. . Reactivity of perovskites with water: role of hydroxylation in wetting and implications for oxygen electrocatalysis. J. Phys. Chem. C 119, 18504−18512 (2015).

[b12] Feng X., Zhai J. & Jiang L. The fabrication and switchable superhydrophobicity of TiO_2_ nanorod films. Angew. Chem. Int. Ed. 44, 5115–5118 (2005). 10.1002/anie.200501337 16021643

[b13] Zhang X. . Preparation and photocatalytic wettability conversion of TiO_2_-based superhydrophobic surfaces. Langmuir 22, 9477–9479 (2006). 17073465 10.1021/la0618869

[b14] Sun T. . Reversible switching between superhydrophilicity and superhydrophobicity. Angew. Chem. Int. Ed. 43, 357–360 (2004). 10.1002/anie.200352565 14705097

[b15] Yu X., Wang Z., Jiang Y., Shi F. & Zhang X. Reversible pH-responsive surface: from superhydrophobicity to superhydrophilicity. Adv. Mater. 17, 1289–1293 (2005).

[b16] Krupenkin T. N., Taylor J. A., Schneider T. M. & Yang S. From rolling ball to complete wetting: the Dynamic tuning of liquids on nanostructured surfaces. Langmuir 20, 3824–3827 (2004). 15969363 10.1021/la036093q

[b17] Meng C., Liu Z., Zhang T. & Zhai J. Layered MoS_2_ nanoparticles on TiO_2_ nanotubes by a photocatalytic strategy for use as high-performance electrocatalysts in hydrogen evolution reactions. Green Chem. 17, 2764–2768 (2015).

[b18] Mohapatra S. K., Misra M., Mahajan V. K. & Raja K. S. Design of a highly efficient photoelectrolytic cell for hydrogen generation by water splitting: application of TiO_2−x_C_x_ nanotubes as a photoanode and Pt/TiO_2_ nanotubes as a cathode. J. Phys. Chem. C 111, 8677–8685 (2007).

[b19] Zhang Q., Hu Z., Liu Z., Zhai J. & Jiang L. Light-gating titania/alumina heterogeneous nanochannels with regulatable ion rectification characteristic. Adv. Funct. Mater. 24, 424–431 (2014).

[b20] Li L. . Underwater superoleophobic porous membrane based on hierarchical TiO_2_ nanotubes: multifunctional integration of oil-water separation, flow-through photocatalysis and self-cleaning. J. Mater. Chem. A 3, 1279–1286 (2015).

[b21] Hu Z. . Regulating water adhesion on superhydrophobic TiO_2_ nanotube arrays. Adv. Funct. Mater. 24, 6381–6388 (2014).

[b22] Balaur E., Macak J. M., Tsuchiya H. & Schmuki P. Wetting behaviour of layers of TiO_2_ nanotubes with different diameters. J. Mater. Chem. 15, 4488–4491 (2005).

[b23] Balaur E., Macak J. M., Taveira L. & Schmuki P. Tailoring the wettability of TiO_2_ nanotube layers. Electrochem. Commun. 7, 1066–1070 (2005).

[b24] Roy P., Berger S. & Schmuki P. TiO_2_ nanotubes: synthesis and applications. Angew. Chem. Int. Ed. 50, 2904–2939 (2011). 10.1002/anie.201001374 21394857

[b25] Shankar K. . Recent advances in the use of TiO_2_ nanotube and nanowire arrays for oxidative photoelectrochemistry. J. Phys. Chem. C 113, 6327–6359 (2009).

[b26] Liu Z., Zhang Q., Zhao T., Zhai J. & Jiang L. 3-D vertical arrays of TiO_2_ nanotubes on Ti meshes: efficient photoanodes for water photoelectrolysis. J. Mater. Chem. 21, 10354–10358 (2011).

[b27] Chen D., Zhang H., Li X. & Li J. Biofunctional titania nanotubes for visible-light-activated photoelectrochemical biosensing. Anal. Chem. 82, 2253–2261 (2010). 20163104 10.1021/ac9021055

[b28] Liu Z., Zhang X., Nishimoto S., Murakami T. & Fujishima A. Efficient photocatalytic degradation of gaseous acetaldehyde by highly ordered TiO_2_ nanotube arrays. Environ. Sci. Technol. 42, 8547–8551 (2008). 19068846 10.1021/es8016842

[b29] Tsubota T., Ono A., Murakami N. & Ohno T. Characterization and photocatalytic performance of carbon nanotube material-modified TiO_2_ synthesized by using the hot CVD process. Appl. Catal. B: Environ. 91, 533–538 (2009).

[b30] Herrmann J., Disdier J. & Pichat P. Photoassisted platinum deposition on TiO_2_ powder using various platinum complexes. J. Phys. Chem. 90, 6028–6034 (1986).

[b31] Wang X. . A mesoporous Pt/TiO_2_ nanoarchitecture with catalytic and photocatalytic functions. Chem. Eur. J. 11, 2997–3004 (2005). 15761909 10.1002/chem.200401248

[b32] Yin H. . Ultrathin platinum nanowires grown on single-layered nickel hydroxide with high hydrogen evolution activity. Nat. Commun. 6, 6430 (2015). 25728293 10.1038/ncomms7430

[b33] Feng X. & Jiang L. Design and creation of superwetting/antiwetting surfaces. Adv. Mater. 18, 3063–3078 (2006).

[b34] Sun T., Feng L., Gao X. & Jiang L. Bioinspired surfaces with special wettability. Acc. Chem. Res. 38, 644–652 (2005). 16104687 10.1021/ar040224c

[b35] Liu M., Zheng Y., Zhai Z. & Jiang L. Bioinspired super-antiwetting interfaces with special liquid-solid adhesion. Acc. Chem. Res. 43, 368–377 (2010). 19954162 10.1021/ar900205g

[b36] Moses P. R. & Murray R. W. Chemically modified electrodes. 3. tin dioxide and titanium dioxide electrodes bearing an electroactive reagent. J. Am. Chem. Soc. 98, 7435–7436 (1976).

[b37] Haensch C., Hoeppener S. & Schubert U. S. Chemical modification of self-assembled silane based monolayers by surface reactions. Chem. Soc. Rev. 39, 2323–2334 (2010). 20424728 10.1039/b920491a

[b38] Lenhard J. R. & Murray R. W. Chemically modified electrodes: part VII. covalent bonding of a reversible electrode reactant to Pt electrodes using an organosilane reagent. J. Electroanal,. Chem. 78, 195–201 (1977).

[b39] Lenhard J. R. & Murray R. W. Chemically modified electrodes. 13. monolayer/multilayer coverage, decay kinetics, and solvent and interaction effects for ferrocenes covalently linked to platinum electrodes. J. Am. Chem. Soc. 100, 7870–7875 (1978).

[b40] Tatsuma T., Tachibana S. & Fujishima A. Remote oxidation of organic compounds by UV-irradiated TiO_2_ via the gas phase. J. Phys. Chem. B 105, 6987–6992 (2001).

[b41] Hu Z. . Photocatalysis-triggered ion rectification in artificial nanochannels based on chemically modified asymmetric TiO_2_ nanotubes. Langmuir 29, 4806–4812 (2013). 23517411 10.1021/la400624p

[b42] Sequeira C. A. C., Santos D. M. F., Šljukić B. & Amaral L. Physics of electrolytic gas evolution. Braz. J. Phys. 43, 199–208 (2013).

[b43] Watt-Smith M. J., Friedrich J. M., Rigby S. P., Ralph T. R. & Walsh F. C. Determination of the electrochemically active surface area of Pt/C PEM fuel cell electrodes using different adsorbates. J. Phys. D: Appl. Phys. 41, 174004 (2008).

[b44] Ralph T. R. . Low cost electrodes for proton exchange membrane fuel cells: performance in single cells and ballard stacks. J. Electrochem. Soc. 144, 3845–3857 (1997).

[b45] Li Y. . Under-water superaerophobic pine-shaped Pt nanoarray electrode for ultrahigh-performance hydrogen evolution. Adv. Funct. Mater. 25, 1737–1744 (2015).

[b46] Lu Z. . Ultrahigh hydrogen evolution performance of under-water “superaerophobic” MoS_2_ nanostructured electrodes. Adv. Mater. 26, 2683–2687 (2014). 24488883 10.1002/adma.201304759

[b47] Faber M. S. . High-performance electrocatalysis using metallic cobalt pyrite (CoS_2_) micro- and nanostructures. J. Am. Chem. Soc. 136, 10053–10061 (2014). 24901378 10.1021/ja504099w

[b48] Wang J., Yang Q., Wang M., Wang C. & Jiang L. Rose petals with a novel and steady air bubble pinning effect in aqueous media. Soft Matter 8, 2261–2266 (2012).

[b49] Shi C. . Interaction between air bubbles and superhydrophobic surfaces in aqueous solutions. Langmuir 31, 7317–7327 (2015). 26065326 10.1021/acs.langmuir.5b01157

