# Supplementary material for: Insight into the Role of Surface Wettability in Electrocatalytic Hydrogen Evolution Reactions Using Light-Sensitive Nanotubular TiO2 Supported Pt Electrodes
Source: Sci Rep. 2017 Feb 6;7:41825. doi: 10.1038/srep41825 (PMC5292970; doi:10.1038/srep41825)
Supplement: Supplementary Information [file srep41825-s1.pdf]

Supplementary information

Insight into the Role of Surface Wettability in Electrocatalytic Hydrogen Evolution Reactions Using Light-Sensitive Nanotubular TiO_2 Supported Pt Electrodes

Chenhui Meng[1], Bing Wang[1], Ziyue Gao[1], Zhaoyue Liu[1,]* Qianqian Zhang[2,]* & Jin Zhai[1,]*

[1] *Key Laboratory of Bio-Inspired Smart Interfacial Science and Technology of Ministry of Education, Beijing Key Laboratory of Bio-inspired Energy Materials and Devices, School of Chemistry and Environment, Beihang University, Beijing 10019, China.*

[2] *School of Physics and Nuclear Energy Engineering, Beihang University, Beijing 100191, China.*

***Corresponding Author. E-mail:** liuzy@buaa.edu.cn; zhangqq@buaa.edu.cn; zhaijin@buaa.edu.cn

Contents:

Figure S1. EDX patterns of OTS-modified Pt-TiO_2-NTs after UV illumination for 200 min on the specific surface of (a) Pt particles and (b) TiO_2 nanotubes. It is found that Si elements mainly distribute on the surface of TiO_2 nanotubes, which indicates that OTS molecules easily bond to TiO_2 nanotubes.

Figure S2. Magnified polarization curves of OTS-modified Pt-TiO_2-NTs at potential ranging -0.08 and 0 V (vs. SHE) when the UV illumination time is 0, 5 and 50 min in 0.5 M H_2SO_4. Following the decrease of water CA by UV illumination, the current density for HERs increases.

Video S1: The video of Pt-TiO_2-NTs with a water CA of ~139 °during polarization process demonstrates that small hydrogen bubbles will merge into a large one, which sticks on the electrode surface strongly.

Video S2: The video of Pt-TiO_2-NTs with a water CA of ~14 °during polarization process indicates that most of large hydrogen bubbles can leave the electrode surface quickly during hydrogen evolution.

Video S3: A gas bubble is easy to adhere onto the surface of hydrophobic Pt-TiO_2-NTs with a water CA of ~139 °because of its large adhesion force.

Video S4: A gas bubble is very hard to adhere onto the surface of hydrophilic Pt-TiO_2-NTs with a water CA of ~14 °because of its extremely low adhesion force.

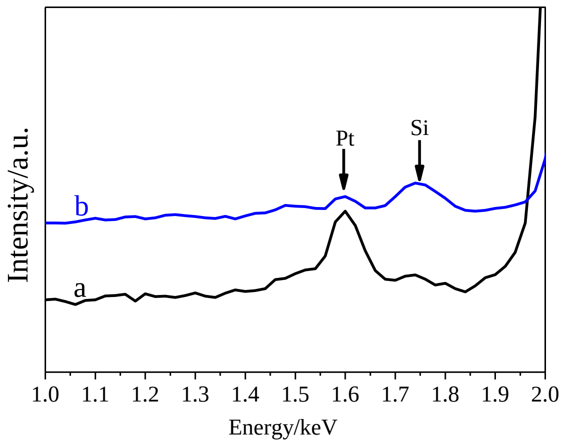

Figure S1. EDX patterns of OTS-modified Pt-TiO_2-NTs after UV illumination for 200 min on the specific surface of (a) Pt particles and (b) TiO_2 nanotubes. It is found that Si elements mainly distribute on the surface of TiO_2 nanotubes, which indicates that OTS molecules easily bond to TiO_2 nanotubes.

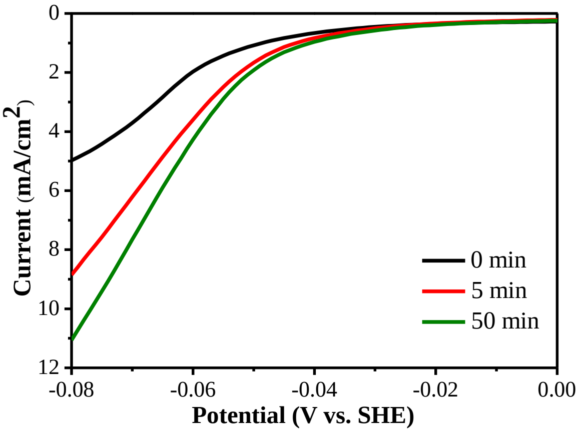

Figure S2. Magnified polarization curves of OTS-modified Pt-TiO_2-NTs at potential ranging -0.08 and 0 V (vs. SHE) when the UV illumination time is 0, 5 and 50 min in 0.5 M H_2SO_4. Following the decrease of water CA by UV illumination, the current density for HERs increases.
